# Text Messaging Based Obesity Prevention Program for Parents of Pre-Adolescent African American Girls

**DOI:** 10.3390/children4120105

**Published:** 2017-12-04

**Authors:** Chishinga Callender, Deborah Thompson

**Affiliations:** USDA/ARS Children’s Nutrition Research Center, Department of Pediatrics, Baylor College of Medicine, 1100 Bates Street, Houston, TX 77030, USA; Chishinga.Callender@bcm.edu

**Keywords:** obesity, prevention, diet, physical activity, parents, African American, text messages, technology, self-determination theory, home environment

## Abstract

African American girls are at a greater risk of obesity than their nonminority peers. Parents have the primary control over the home environment and play an important role in the child obesity prevention. Obesity prevention programs to help parents develop an obesity-preventive home environment are needed. The purpose of this study was to collect formative research from parents of 8–10-year old African American girls about perceptions, expectations, and content for a text messaging based program. Mothers (*n* = 30) participated in surveys and interviews to inform message development and content. A professional expert panel (*n* = 10) reviewed draft text messages via a survey. All the mothers reported owning a cellphone with an unlimited texting plan, and they used their cellphones for texting (90.0%) and accessing the Internet (100.0%). The majority were interested in receiving text messages about healthy eating and physical activity (86.7%). Interviews confirmed survey findings. One hundred and seven text messages promoting an obesity-preventive home environment were developed. The expert panel and parents reported positive reactions to draft text messages. This research provides evidence that mobile health (mHealth) interventions appeal to parents of African American girls and they have ready access to the technology with which to support this approach.

## 1. Introduction

African American girls are at greater risk of obesity than their white peers. Between the ages of 6 to 19 years old, the prevalence of obesity was 26% in African American girls compared to 16% in non-Hispanic white girls [1]. Obese children are at risk of health complications, such as hypertension and type 2 diabetes [2], and of becoming obese adults [3]. Obesity is a result of a long-term energy imbalance, where energy intake exceeds expenditure [4]. Energy balance is dependent on diet and physical activity [5,6]. African American girls’ diet and physical activity behaviors may put them at an increased risk for obesity [7,8,9]. Modifying dietary and physical activity behaviors can reduce the risk of obesity and related complications [10]. Because childhood diet [9] and physical activity [4] behaviors are often continued into adulthood, it is important to establish healthy behaviors at an early age. In addition to diet and physical activity, sedentary behavior, stress, and sleep have been identified as risk factors for childhood obesity [11,12,13]. Thus, healthy sleep patterns and regulating sedentary behavior and stress are also essential behaviors that need to be established at an early age to reduce obesity risk.

Parents have primary control over the home environment [14]. Therefore, they are an important component of child obesity prevention. It is critical for parents to encourage nutrition and physical activity behaviors at an early age to reduce children’s risk of obesity and related chronic diseases and to help them develop and maintain a healthy lifestyle. There is evidence that parental practices regarding diet and physical activity differ for girls and boys [15,16,17,18]. Thus, tailoring obesity prevention programs by gender may improve their effectiveness [19]. Several obesity prevention programs have been developed for African American girls promoting these behaviors [20,21,22,23,24,25,26,27,28,29,30], but few have been developed with a specific focus on their parents as the agent of change [31,32,33,34]. Intervention programs, specifically designed for African American families, are needed to promote healthy eating and physical activity at an early age in culturally appropriate ways. It is imperative that obesity prevention programs are culturally appropriate to increase likelihood of success [35].

Technology based intervention programs may be a feasible approach to promoting healthy eating and physical activity behaviors. Cell phones and the Internet are widely used by African Americans. According to the Pew Center, 92% of African Americans own a cell phone and 56% own a smartphone [36]. Further, 80% of African Americans use the Internet [36]. Research has shown that 53% of African American parents used the Internet to seek health information [37]. Furthermore, text messaging is widely used by African Americans; 79% of African Americans send and receive text messages [38]. In a recent study, 87.4% of African American mothers were interested in receiving health information by mobile phones [37]. In a study with obese women, mostly African American, 70% reported that receiving health related text messages were easy and helpful [39]. In a study with African American women, participants in the Facebook and text message intervention group reported gaining knowledge (93%) from the text messages and the texts were helpful for promoting physical activity (79%) [40]. Previous studies have explored Internet usage, cell phone usage, and text messaging in developing health based interventions for African American parents [37]. However, studies have not explored the use of text messaging in the development of a parent-focused obesity based prevention program for pre-adolescent African American girls.

Interventions should be guided by psychological theories [41]. The self-determination theory (SDT) is a theory of motivation and posits that three basic psychological needs dictate motivation to engage in a particular behavior: (1) competence (i.e., knowledge, skills, ability); (2) autonomy (i.e., choice, control); and (3) relatedness (i.e., connection to self and others) [42]. Fulfilling these needs helps integrate the behavior with one’s self-identify and self-definition. The higher the degree to which these needs are met, the greater the need satisfaction, resulting in autonomous (self-directed) motivation to engage in the behavior, which often results in greater short- and long-term behavioral performance [42].

The purpose of this study was to collect formative research to identify (1) perceptions of parents of 8–10-year old African American girls about receiving text messages promoting a healthy home food and activity environment; (2) expectations regarding program content; (3) familiarity, use, and availability of technology needed to participate in a program of this type; and (4) comments and suggestions regarding text message content and structure.

## 2. Materials and Methods

### 2.1. Design

A mixed methods approach (surveys, telephone interviews) was used to collect data with which to develop the text messages. The protocol was approved by the institutional review board at Baylor College of Medicine (H-27505).

### 2.2. Study Participants

Two participant groups were recruited. The first group consisted of parents (“parents”), while the second consisted of health professionals (“expert panel”). It was thought that this approach would help ensure the text messages were appealing, as well as scientifically accurate.

Parent inclusionary criteria included being the parent of an 8–10-year-old African American girl, having access to a mobile phone that sends and receives text messages, and a willingness to receive text messages from the study. Exclusionary criteria included lack of access to a mobile phone that sends and receives text messages and an unwillingness to receive and send text messages. Parents provided written informed consent prior to participation.

Inclusionary criteria for the expert panel included being a health professional who was of African American descent and/or who had conducted research with African American children and/or parents. Exclusionary criteria included a health professional not of African American descent and/or lack of experience conducting research with African American children and/or parents. Data collection was anonymous; therefore, informed consent was not required.

### 2.3. Sample Size

The literature offers a lack of direction on appropriate sample sizes for formative research. Therefore, the sample sizes were based on the concept of theoretical saturation, or the point at which no new information was gained [43]. Our goal was to recruit 30 parents and 10 health professionals. Based on an examination of the data, theoretical saturation was attained with both samples.

### 2.4. Recruitment

Parents were recruited from the volunteer database at the Children’s Nutrition Research Center (CNRC). The study coordinator contacted parents of 8–10-year old African American girls who had agreed to be included in the database and were interested in participating in research studies. She informed parents about the study, described it in detail, and screened interested parents for eligibility. The expert panel was recruited verbally and through email. They were informed about the study in detail and asked if they would be interested in participating on the panel. If they agreed, they were included on the panel.

### 2.5. Data Collection

Parents participated in two rounds of formative research. During each round, they completed an online survey, followed by a telephone interview to discuss survey responses and obtain additional information. Parents were emailed a link and private password to complete each survey. The expert panel completed one online survey. They received a link to complete the online survey anonymously. Surveys were hosted on a secure website.

#### 2.5.1. Phase 1: Parents

Similar to previous research, in the first phase of data collection, parents completed a 50-item online survey developed by the team to identify their use of mobile and Internet technology (e.g., Internet, cell phone, text messaging) and beliefs, values, and practices related to diet, physical activity, sedentary behavior, and body weight [44]. Sample items included “How often do you send or receive text messages”, “Would you like to receive text messages about healthy eating or physical activity”, and “How important is it for children to eat healthy”.

A trained interviewer then conducted a semi-structured telephone interview with each parent to discuss their responses and to obtain more information and insight with which to develop the text messages. The interviews were scripted and contained open-ended, non-leading questions; probes and prompts were used to clarify and explore responses. Examples of interview questions included “What are examples of factors that make it hard for families to help their children make healthy food and physical activity choices at home”, “How should healthy eating tips be incorporated into the text messages”, and “How important are cultural influences on the foods your family eats at home”. Each interview was digitally recorded and designed to take no more than one hour to complete. Based on this information, the research team developed 107 text messages informed by: (1) the Self-Determination Theory (i.e., specifically promoting the basic psychological needs—autonomy, competence, relatedness—to enhance autonomous motivation) [42], and (2) the five behaviors promoting an obesity-preventive home environment (healthy eating, physical activity, sedentary behavior, sleep patterns, stress).

#### 2.5.2. Phase 2: Expert Panel

An expert panel, comprised of health professionals, was invited by email to review 107 text messages in an anonymous online survey. The panel was asked to review the messages for scientific accuracy, cultural appropriateness, and practicality using a 2-item response format (modify, eliminate). The text messages were modified based on the comments and suggestions provided by the expert panel.

#### 2.5.3. Phase 3: Parents

In the third phase of data collection, parents reviewed the revised text messages to assess acceptability. They were asked to consider whether the texts would be helpful, realistic, and culturally appropriate using a 3-item response format (appropriate, modify, eliminate). A follow up telephone interview was conducted to further discuss the text messages (i.e., categories, length, website links, emoticons), reasons why they chose to modify or eliminate a text message, and to obtain additional feedback with which to modify the text messages to enhance acceptability. Sample interview questions included “Overall, what did you think about them [text messages]”, and “If you received a text message with a link to more information, would you click on the link”. Rating scales were also used: for example, during the interview, mothers were asked “Using a scale of 1 to 5 (1 = very unlikely, 2 = a little unlikely, 3 = not likely or unlikely, 4 = a little likely, 5 = very likely), how likely would you share the text messages with family members and/or friends”. Each interview was designed to take no more than one hour to complete.

### 2.6. Data Analysis

Survey responses were descriptively analyzed using Statistical Analysis Software (SAS) (version 9.4, SAS Institute Inc., Cary, NC, USA, 2010). Frequencies and percentages were calculated for survey responses. After each interview, summaries of key points that emerged from each round of interviews were generated and used to inform text message content, design, and needed revisions [45]. Verbatim quotes were used to support qualitative findings.

## 3. Results

### 3.1. Participant Characteristics

Recruitment for the study began in October 2014 and ended in January 2015. Thirty mothers of 8–10-year old African American girls enrolled in the study. Mothers were African American (100%), 31–50 years old (86.6%), married (50%), and all had at least one 8–10-year old daughter. All completed the first phase of the study. Phase one interviews lasted an average of 45 min. Two mothers did not participate in the second phase of the study as a result of an inability to contact them via phone, email, or mailing address. Ten diverse expert panel members were recruited and participated in the study. The expert panel consisted of 4 health research professionals, 3 behavioral scientists, 2 pediatricians, and 1 clinical psychologist. All met inclusionary criteria.

### 3.2. Phase 1: Parent Survey Results

#### 3.2.1. Cell Phone Access

All mothers reported using cell phones, having an unlimited text messaging plan, and most had an unlimited data plan (86.7%) (data not shown). All also reported using their mobile phones to make calls and access the internet. Common mobile phone activities included email (96.7%), taking photos (96.7%), texting (90.0%), playing games (83.3%), and downloading/playing apps (76.7%). Most of the mothers reported that they sent or received texts daily (93.3%) (Table 1).

#### 3.2.2. Internet Usage

Mothers were regular users of the Internet. When asked how they accessed the Internet, cell phones were the top response (100%), followed by the computer (96.7%), and the tablet (70%). Most reported accessing the Internet from home (96.7%), followed by work (86.7%), and their car (60%). The five most common types of information accessed were entertainment (90%), work (83.3%), news (83.3%), educational information (83.3%), social media (80%), and finances (70%). Some mothers also sought health information on the Internet (66.7%) (Table 2).

#### 3.2.3. Obesity Prevention Text Messages

The majority of mothers were interested in receiving text messages about healthy eating and physical activity (86.7%). They thought this would be a good way to give parents tips on ways to help their daughters eat healthy foods (96.7%) and be physically active at home (93.3%). Most preferred receiving health-oriented text messages in the morning (56.7 %), while some preferred afternoon (23%) or evening (20%) texts. Preferred frequency was 2 or 3 times a week (data not shown).

### 3.3. Phase 1: Parent Interviews

The interviews confirmed that the mothers were technology savvy and were avid users of mobile phones, texting, and the Internet. They also confirmed that mothers were interested in receiving short, informative text messages about healthy eating and physical activity to promote a healthy home and food activity environment for their daughters.

The interviews provided important insights into what the text messages should include to make them relevant, practical, and appealing to busy families. A key point shared by the mothers was that establishing a foundation of healthy behaviors at home was a key family value. Several reasons mentioned by mothers on the importance for children to eat healthy and be physically active included “to learn habits early on”, “improves quality of life”, and “to keep them from [becoming] obese”. They saw parents as the “first teachers and guiders”, who must set the primary example for their child to develop and practice healthy eating and physical activity behaviors. One mother mentioned that “parents play a significant role and example”. The mothers also emphasized the need for easily accessible resources and tips to help them create a healthy home environment. One mother recommended text messages that would “introduce foods in creative ways for the girls to be involved” and “fun things for the both parents and girls to do together”.

While time, convenience, costs, needs of children, and activities outside the home were identified as the major challenges to healthy eating and physical activity, differing views emerged regarding the role culture played in healthy behaviors. When mothers were asked “how important are cultural influences on the foods your family eats at home”, some mothers stated that individual choice, not culture, influenced foods eaten at home; alternatively, others said that while culture influenced foods eaten at home, they chose to prepare traditional foods in healthier ways. One mother stated, “culture shouldn’t be an excuse; healthy eating and physical activity should be encouraged in all cultures”. Another mother stated, you can “still make cultural meals healthy and fresh”. Examples of cultural influences given on foods eaten at home were Sunday dinners and holiday meals. Similarly, when mothers were asked, “how important are cultural influences on whether your family is physically active”, some did not believe culture influenced physical activity; others shared that culture may influence family activity level depending on exposure to physical activity and having a model in the home for being physically active. For example, one mother shared that growing up her “mom was always active in sports, and her children saw her doing that”. Another mother shared that “it is not a culture issue, it is a healthy issue”.

### 3.4. Text Message Development

**Figure 1:**
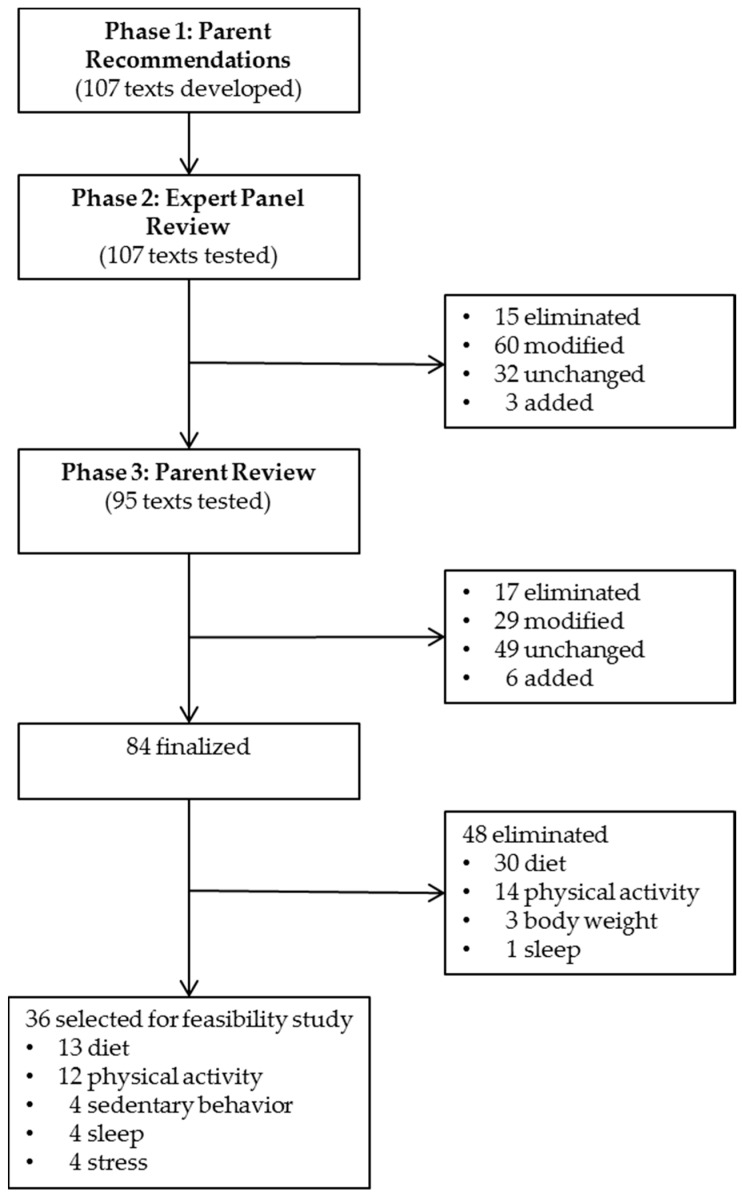
Text Message Development Phases 1–3.

Using information obtained from mothers, 107 text messages were developed by the research team (Figure 1). The majority of the text messages emphasized ways in which to modify the home environment to promote and support healthy diet and physical activity. Because sedentary behavior, stress, and sleep may also influence obesity risk in children [11,12,13], text messages were also developed to promote these behaviors.

The text messages were designed to satisfy the basic psychological needs (autonomy, competence, relatedness) [42] (Table 3). They were <160 characters, had a positive tone, and most contained links to reputable websites (e.g., ChooseMyPlate.gov, EatRight.org, Health.gov).

### 3.5. Phase 2: Expert Panel

Text messages (*n* = 107) were then reviewed by the ten-member expert panel. Comments provided suggestions on how to modify the messages, reasons to eliminate a message, and/or overall thoughts and suggestions on ways to refine the messages. Of the 107 text messages that were reviewed, 43 received no recommendations to modify or eliminate, and 47 were rated as needing modification. The remaining text messages were rated as eliminate (*n* = 4) or both modify and eliminate (*n* = 13) (Table 4). Examples of suggested modifications are presented in Table 5.

Of the 107 texts reviewed, 15 were eliminated based on expert panel review and 92 were retained. Of the 92 texts retained, 60 were modified, while 32 were unchanged (Figure 1). Three new text messages were added with a diet focus based on suggestions by the expert panel. Thus, 95 text messages were finalized for phase 3. The 95 text messages were distributed as follows: 43 diet; 34 physical activity; 6 sleep; 4 sedentary behavior; 4 body weight; 3 stress; 1 diet and physical activity. Of these SDT grounded text messages (*n* = 95), 22 promoted autonomy, 44 competence, and 29 relatedness.

### 3.6. Phase 3: Parent Survey Results

All 95 text messages were rated as “appropriate”. However, only 23 were rated as not needing modification and/or elimination. For the remaining 72 text messages, 40 were also rated as “modify”, 5 as “eliminate”, and 27 as “modify and eliminate” (Table 6).

Ratings and comments were reviewed to identify ways to make the texts appealing and relevant to mothers. Of the 95 texts, 17 were eliminated and 78 were retained. Of the 78 texts retained, 29 were modified (Figure 1). Six new text messages were added on diet, including texts on the benefits of healthy eating (i.e., “Share the benefits of eating grains with your family”). Thus, a total of 84 text messages were finalized for use in the 12 feasibility week study (one text per day). The 84 text messages were distributed as follows: 43 diet; 24 physical activity; 5 sleep; 4 sedentary behavior; 4 body weight; 3 stress; 1 diet and physical activity. Of these SDT grounded text messages (*n* = 84), 18 promoted autonomy, 37 competence, and 29 relatedness. Examples of modifications after the texts were reviewed by the parents are presented in Table 7.

Parents suggested receiving at most 3 messages each week rather than daily text messages. Thus, 36 of the 84 text messages were selected for the study, distributed as follows: 13 diet, 12 physical activity, 4 sedentary behavior, 4 sleep, and 3 stress (Figure 1).

### 3.7. Phase 3: Parent Interviews

The interviews confirmed that the mothers were interested in receiving text messages on all five behaviors (diet, physical activity, sedentary behavior, stress, and sleep) related to risks of childhood obesity. Overall, the mothers thought the text messages were informative, helpful, good reminders, and provided realistic ideas. One mother shared, “I thought they were pretty good, seemed to be well thought out, and really informative”. They also thought the text messages were a good length—i.e., were not too long or too short. They also liked that some of the texts contained emoticons.

Mothers also liked the website links provided in the text messages. They were described as being “very informative”, “a reminder”, “an introduction to more information”, and “very helpful to share with their children”. All mothers said they would click on the link if they were to receive a text message with one. Overall reasons for clicking on the link included they were a source of more information and provided convenience and choice. One mother shared, “I love the links. It was easy access to get the information right in front of you”.

Mothers were open to receiving a weekly email summary with the text messages sent that week. Some preferred clicking on the message link in an email, as it would be easier to access, enable viewing the link on the computer, and give them the opportunity to refer back to the link. One mother shared, “More likely email because I can sit and read it through. I would open it in the text, but to actually get into it more likely [email]”. Some, however, preferred clicking on the link in the text message as it would be easier and more convenient for them. Another mother shared her reason for clicking on the link in the text, “Because it will pop right up on my phone whereas the emails, I have 50 million emails”. Some mothers also preferred being able to click on the links in the email and text message. One mother shared, “Both actually … because either way if it’s in an email, I can click on the link; if it’s in a text, I still do. But sometimes you may have people where they are going to read the message depending on the time of day that message comes in. Let’s say they’re at work, then in the email, they’re just more prone to go back and see the link there”.

All the mothers said their child and/or family would be interested in trying the tips and activities from the links within texts promoting diet or physical activity; the main reason for saying yes was to try “something new and different”. When asked how likely they would be to share the text messages with family members and/or friends most reported they would be “most likely” to do this. Reasons included a desire for their family and friends to be healthy, having family and friends with children who would benefit from the information, and liking the idea of sharing the information.

## 4. Discussion

The purpose of this study was to explore the preferences and expectations of mothers of 8–10-year old African American girls regarding text messages promoting a healthy home food and activity environment. The study also sought to identify their technology availability, use, and familiarity and reactions to text messages promoting a healthy home environment that promotes and supports obesity prevention. Findings revealed that African American adults, specifically mothers, are avid users of cell phones and the Internet, and text messages promoting a healthy home food and activity environment were an appealing way to receive health-oriented messages.

Previous literature has reported on African American parents using cell phones and the Internet. Similar to our study, the Pew Research Center data found that the majority of African Americans are internet users and cell phone owners [36]. Furthermore, a survey measuring cell phone and Internet usage reported that the majority of African American parents own cell phones and used their phones to access the Internet [37].

Mothers in our study were avid users of text messaging. This is similar to findings by previous reports and studies. Pew Research Center data found that the majority of African Americans send or receive text messages [38]. As in our study, a study by Mitchell et al. found that the majority of African American parents use their cell phones for text messaging [37]. It also reported that a majority of the parents had a plan with unlimited texting and data [37]. These findings were similar to our sample, with all of the mothers reporting they had unlimited texting and the majority reporting they had unlimited data.

Mothers in our study were interested in receiving text messages on healthy eating, physical activity, sedentary behavior, sleep, and stress. They thought text messages would be a helpful way to receive this information because texts are convenient, easily accessible, and provide a constant reminder. This is similar to findings by Mitchell et al. [37], which found that more than 87% of African American parents, primarily mothers, were interested in receiving health information online through email or texts.

Although text messages promoting healthy lifestyle behaviors were viewed favorably by parents, they cautioned that the text messages should be short, easy to understand, and provide helpful tips. This is similar to findings by Pagan et al. [46], which found that adolescent and young adult African American women preferred text messages that were brief and included dietary and physical activity tips. Mothers reported wanting links to websites included in the text messages as a source for additional information. The favorable feedback, including that the messages were informative and helpful, is similar to findings in a recent study done with African American women in an intervention promoting physical activity. In that study, women reported that text messages were a helpful way to promote physical activity and that they gained knowledge from the texts [40]. In a text messaging based intervention for adults with diabetes promoting adherence to self-management behaviors, participants reported high levels of satisfaction with the program and most agreed that it was easy to use and helped with diabetes self-care [47]. Steinberg et al. found that a text messaging based intervention helped obese women, mostly African American, to increase the number of steps walked daily [39]. Those receiving daily text messages reported that they were very important [39]. These findings provide insight into how to develop a text messaging based program that addresses the needs and interests of parents.

The formative research for the study was conducted to provide insight into intervention content and structure of a text message based intervention designed to help parents of 8–10-year old African American girls develop a healthy home food and activity environment in order to reduce obesity risk. There is an emerging body of literature that supports the wide use of technology by African Americans. African American families have access to technology and are open to the idea of receiving text messages promoting a healthy home food and activity environment.

This study explored the perceptions of mothers with 8–10-year old African American girls regarding text messages specifically geared towards creating a healthy home environment to promote and support child obesity prevention. Strengths of this study included scripted interviews, an interviewer trained in qualitative methods, and use of a mixed methods methodology. The limitations included a small sample size and the use of only one geographic location in the United States, which limits generalizability. Despite the limitations, the findings provide an opportunity to develop a text messaging based intervention addressing the interests and needs of the mothers.

Future research should test the texts messages to first examine feasibility, then the effect on the home environment and child behavior (i.e., dietary choices, increased physical activity). Future research should also explore cell phone usage and Internet access among African American parents in different geographic regions to identify factors that may influence acceptability and use of a technology based intervention in a broader population. It may be that parents in different regions have different access than parents in this study. Future research is also needed to assess content preferences of parents in different geographic regions; it may be that parents in other areas have different preferences regarding text message content and focus. Additionally, future research is needed to explore the role of culture on parenting practices regarding food and physical activity and how to incorporate this into text messages designed specifically for parents of African American girls. Finally, future research could explore the cross-cultural universality of the text messages, and identify what modifications, if any, would be needed to ensure appropriateness for parents of other racial and ethnic groups. Addressing these needs in future research will help to develop a convenient and accessible mobile health (mHealth) intervention that has broad appeal.

## 5. Conclusions

This study highlighted that parents of African American girls have access to technology, use mobile technology and the Internet frequently, and are interested in receiving text messages on creating a healthy home environment that promotes and supports child obesity prevention. Working with parents to develop program content may be a broadly dispersible method for reducing disparities related to child obesity and provide access to obesity prevention programs by developing a convenient and accessible method for increasing healthy lifestyle behaviors in African American girls.

## Figures and Tables

**Table 1:** Descriptive statistics for cell phone usage (*n* = 30).

	*n*	Percentage
**What Do You Use Your Cell Mobile Phone For?**		
Making calls	30	100.0
Accessing the Internet	30	100.0
Email	29	96.7
Taking photos	29	96.7
Texting	27	90.0
Playing games	25	83.3
Downloading/playing apps	23	76.7
Other	4	13.3
**How Often Do You Send or Receive Text Messages on Your Mobile Phone?**		
Daily	28	93.3
Weekly	2	6.7

**Table 2:** Descriptive statistics for Internet usage (*n* = 30).

	*n*	Percentage
**Please Check All the Ways You Access the Internet**		
Cell phone	29	100.0
Computer	30	96.7
Tablet	21	70.0
Other	2	6.7
**Where (i.e., Location) Do You Access the Internet? Please Check All That Apply**		
Home	29	93.3
Work	26	86.7
Car or other vehicle	18	60.0
School	7	23.3
Community center	3	10.0
Library	2	6.7
**What Type of Information Do You Usually Access on the Internet?**		
Entertainment	27	90.0
Work	25	83.3
News	25	83.3
Educational	25	83.3
Social media	24	80.0
Finances	21	70.0
Health	20	66.7
Religious	19	63.3
School	17	56.7
Other	2	6.7

**Table 3:** Sample dietary messages based on self-determination theory.

SDT Category	Text Message
Autonomy	“Reduce the fat in your family’s diet. Choose 1% or 2% reduced milk instead of whole”
Competence	“Check out these recipes for quick and easy family meals”
Relatedness	“Ask your child to help you choose vegetables at the grocery store. Check out this link on choosing fresh fruit and veggies”

SDT: Self-determination theory.

**Table 4:** Expert panel survey summary of text messages (*n* = 107 texts).

Survey Response	Number of Text Messages
No response *	43
Modify	47
Eliminate	4
Both (Modify and Eliminate)	13

* Refers to text messages that were not selected for modification or elimination.

**Table 5:** Sample messages after expert panel review.

Original Message	Expert Panel Suggestions	Revision
“Make the choice to use healthy oils when preparing family meals. Check out the facts here”	Delete “Make the choice”; begin the sentence with “Use healthy”	“Want to help your family eat healthy? Use healthy oils when preparing meals. Check out the facts here”
“Long day? Make the choice to prepare on-the go-snacks ahead of time”	“Make the choice” is too repetitive	“Long day? Prepare healthy, on-the-go snacks ahead of time”
“Make the choice to limit your children’s computer time”	Add “No more than 2 h a day of screen time”; What if a parent only has one child?	“Help your child be less inactive. Limit their screen time to no more than 2 h a day. Get up and get moving instead!”

**Table 6:** Parent survey summary of text messages (*n* = 95 texts).

Survey Response	Number of Text Messages
Appropriate *	23
Modify	40
Eliminate	5
Both (Modify and Eliminate)	27

* Refers to text messages that were selected as appropriate by all parents (100%).

**Table 7:** Sample messages after parent review.

Original Message	Parent Suggestions	Revision
“Cut the fat! Replace sour cream with plain low-fat yogurt. Take charge, and make the change!”	Fat is too aggressive; Soften the message by replacing “Cut the fat” with “Reduce the fat”	“Reduce the fat. Try low fat sour cream or yogurt when preparing meals. Take charge, and make the change”
“Add variety to family meals. Serve a meatless meal once a week”	Change the wording of the message	“Try a meatless meal once a week”
“Carrots and celery with hummus or low fat ranch dip make a tasty afternoon snack for your child and family”	Dislike hummus; Alternative snack such as peanut butter and apples	“Carrots and celery with low fat peanut butter or low fat ranch dressing make a tasty snack for your child and family”
“Ask everyone in the family to wear a pedometer, and see who gets the most steps each week!”	Include information on what a pedometer is	“Ask everyone in the family to wear a pedometer, a device that counts your steps as you walk, and see who gets the most steps each week!”
“Help your child be less inactive. Limit their screen time to no more than 2 h a day. Get up and get moving instead!”	Simplify and change the wording of the message	“Help your child be more active. Limit screen time to no more than 2 h a day”
